# Responses of symbiotic nitrogen-fixing common bean to aluminum toxicity and delineation of nodule responsive microRNAs

**DOI:** 10.3389/fpls.2015.00587

**Published:** 2015-07-30

**Authors:** Ana B. Mendoza-Soto, Loreto Naya, Alfonso Leija, Georgina Hernández

**Affiliations:** Functional Genomics of Eukaryotes, Centro de Ciencias Genómicas, Universidad Nacional Autónoma de MéxicoCuernavaca, Mexico

**Keywords:** common bean, aluminum toxicity, symbiotic nitrogen fixation, legume-rhizobia, nodules, microRNAs

## Abstract

Aluminum (Al) toxicity is widespread in acidic soils where the common bean (*Phaseolus vulgaris*), the most important legume for human consumption, is produced and it is a limiting factor for crop production and symbiotic nitrogen fixation. We characterized the nodule responses of common bean plants inoculated with *Rhizobioum tropici* CIAT899 and the root responses of nitrate-fertilized plants exposed to excess Al in low pH, for long or short periods. A 43–50% reduction in nitrogenase activity indicates that Al toxicity (Alt) highly affected nitrogen fixation in common bean. Bean roots and nodules showed characteristic symptoms for Alt. In mature nodules Al accumulation and lipoperoxidation were observed in the infected zone, while callose deposition and cell death occurred mainly in the nodule cortex. Regulatory mechanisms of plant responses to metal toxicity involve microRNAs (miRNAs) along other regulators. Using a miRNA-macroarray hybridization approach we identified 28 (14 up-regulated) Alt nodule-responsive miRNAs. We validated (quantitative reverse transcriptase-PCR) the expression of eight nodule responsive miRNAs in roots and in nodules exposed to high Al for long or short periods. The inverse correlation between the target and miRNA expression ratio (stress:control) was observed in every case. Generally, miRNAs showed a higher earlier response in roots than in nodules. Some of the common bean Alt-responsive miRNAs identified has also been reported as differentially expressed in other plant species subjected to similar stress condition. miRNA/target nodes analyzed in this work are known to be involved in relevant signaling pathways, thus we propose that the participation of miR164/NAC1 (NAM/ATAF/CUC transcription factor) and miR393/TIR1 (TRANSPORT INHIBITOR RESPONSE 1-like protein) in auxin and of miR170/SCL (SCARECROW-like protein transcription factor) in gibberellin signaling is relevant for common bean response/adaptation to Al stress. Our data provide a foundation for evaluating the individual roles of miRNAs in the response of common bean nodules to Alt.

## Introduction

Legumes are second only to the Graminiae in their importance to humans. Grain legumes provide more than one–third of humankind nutritional nitrogen requirements. A hallmark trait of legumes is their ability to establish mutualistic symbioses with nitrogen-fixing bacteria collectively known as rhizobia. Symbiotic nitrogen fixation (SNF) by differentiated bacteroids takes place in specialized rhizobia-induced root nodules. This process involves a tight association between the two symbionts. SNF reduces the cost of legume cultivation and makes them valuable source of soil nitrogen to other crops (Graham and Vance, 2003).

Common bean (*Phaseolus vulgaris*) is the most important legume for human consumption. In Mexico and other countries common bean are staple crops serving as the primary source of protein in the diet. Common bean is mainly grown by small landholders in tropical areas of Latin America and Africa; soil acidity in the tropics is a major constraint for crop productivity (Broughton et al., 2003; Graham et al., 2003). It has been estimated that almost 50% of the world’s potentially arable lands are acidic and the American continent accounts for 40% of the world’s acid soils (von Uexküll and Mutert, 1995). Poor crop growth in acid soils is due usually to a combination of metal toxicity and nutrient deficiency, primarily toxic levels of aluminum (Al) and manganese (Mn) and suboptimal levels of phosphorus (P) (von Uexküll and Mutert, 1995; Kochian et al., 2004). Research from our group, based on transcriptomics, metabolomics, and miRNA profiles, has contributed to define the response of common bean to P deficiency and to Mn toxicity (Hernández et al., 2007, 2009; Valdés-López et al., 2008, 2010; Ramìrez et al., 2013). In this work we analyze the response of SNF common bean to Al toxicity (Alt), an important constraint for common bean crop production in Mexican acidic soils.

Al toxicity is the primary growth-limiting factor in acidic soils. Solubility of Al is pH-dependent, at soil pH values below 5.5 Al^3+^ is solubilized into soil solution and this is the most important rhizotoxic Al species. High levels of Al^3+^ in the soil inhibit root growth and function, increase the risk of plants to succumb to drought and to mineral deficiencies and reduce crop production (Kochian et al., 2004). Considerable advances have been made to understand the mechanisms of Alt, but some aspects remain unclear (reviewed by Kochian et al., 2005; Vitorello et al., 2005). Some Alt symptoms and responses are detectable shortly (seconds or minutes) after Al exposure, while others are only discernable after long-term (hours or days) exposure. The timing and type of plant responses to Alt shows high variability among plants species/genotypes, different experimental conditions used for analysis or to diverse natural/environmental conditions for crop production (Kochian et al., 2005). The primary and earliest symptom of Alt is a rapid inhibition of root growth and lateral root formation; Al disrupts root cell expansion and elongation leading to inhibition of cell division (Frantzios et al., 2001). A recent detailed analysis by Kopittke et al. (2015) identified that the primary lesion of Al is apoplastic, reducing root growth at very short period by binding to the walls of outer cells and directly inhibiting their loosening in the elongation zone. The root apex is considered to be the primary target of Al stress, where Al affects diverse cellular processes and signal-transduction pathways due to its high reactivity. Al has strong affinity to negatively charged plasma membrane, thus can modify membrane structure and cause depolarization. In addition, Al ions can displace other cations that may form bridges between the phospholipid head groups of the membrane bilayer altering membrane fluidity and homeostasis, leading to disturbance of ion-transport processes (Akeson et al., 1989; Lindberg et al., 1991). Al induces accumulation of reactive oxygen species (ROS) that cause peroxidative damage to lipids and lead to mitochondrial dysfunction that could be related to root growth inhibition (Yamamoto et al., 2001). Oxidative damage leads to a disturbance of cellular homeostasis that may result in cell death; Alt induced cell death has been observed in the elongation zone of roots from metal-stressed plants (Yamamoto et al., 2003). After analyzing the response to Alt of the legume *Lotus corniculatus*, Navascués et al. (2011) concluded that oxidative stress is a consequence not a cause of Alt. The apoplast accumulation of the polysaccharide callose has also been documented as a characteristic symptom of Alt in plants, it may lead to cellular damage by inhibiting intercellular transport through plasmodesmatal connections and to cell wall rigidification that promotes growth inhibition of the root (Horst et al., 1997; Sivaguru et al., 2000). Clear evidence from different plant genotypes indicates that a mechanism for Al tolerance is Al-exclusion based on carboxylates exudation form roots apex; Al-carboxylate complexes are not transported into roots or across membranes (Kochian et al., 2005; Vitorello et al., 2005).

Acidity and high Al in tropic and temperate soils pose an additional challenge for legumes because their symbiotic rhizobia are sensitive to acidity. Reduced growth in acidic/Alt condition has been observed in different rhizobial species, both in laboratory conditions and in natural environments (Graham et al., 1994; O’Hara and Glen, 1994; Paudyal et al., 2007; Avelar Ferreira et al., 2012). Root nodule bacteria can be more sensitive to low pH and Alt than their legume host, and so their survival and persistence in acidic soils results in diminished infection, nodulation and SNF. Total or partial nodulation inhibition in legumes exposed to high Al has been reported for several species such as common bean (*P. vulgaris*), clover (*Trifolium repens*), *Stylosanthes* species and other tropical legumes (De Carvalho et al., 1981; Franco and Munns, 1982a; Wood et al., 1984; Paudyal et al., 2007).

Regulatory mechanisms for plant adaptation to metal toxicity and other stresses involve microRNAs (miRNAs) along with other regulators. miRNAs are 21–24 nt-long non-protein-coding RNAs that regulate plant gene expression at the posttranscriptional level through the transcript cleavage or translation inhibition of their specific mRNA target(s). Generally, miRNA target genes code for transcription factors, stress response proteins, and other proteins that impact the development, growth, and physiology of plants. This mechanism operates through the recruitment of a miRNA-containing effector complex, that includes ARGONAUTE 1 (AGO1) protein, to its target mRNA by base-pairing complementarity (Rogers and Chen, 2013). In addition, miRNAs (23–24 nt-long), loaded to AGO4, are capable of transcriptional gene silencing by triggering DNA-methylation at some of their target sites (Chellappan et al., 2010; Rogers and Chen, 2013). Several reports have shown the role of miRNAs in the response/adaptation of plants to different abiotic stresses including metal toxicity (reviewed by Gielen et al., 2012; Kraiwesh et al., 2012; Mendoza-Soto et al., 2012; Sunkar et al., 2012; Gupta et al., 2014; Zeng et al., 2014). Specifically, recent studies based in high-throughput sequencing technology, genome-wide analysis of small RNAs and degradome have identified root miRNAs that respond to high Al levels (reviewed by Yang and Chen, 2013; He et al., 2014). These include Alt-responsive miRNAs from rice (*Oryza sativa* sp *indica* and *O. sativa* sp *japonica*; Lima et al., 2011) and from the legumes *Medicago truncatula* (Zhou et al., 2008; Chen et al., 2012) and wild soybean (*Glycine soja*; Zeng et al., 2012). Tobacco (*Nicotiana tabacum*) miRNAs that respond to aluminum oxide (Al_2_O_3_) nanoparticles have also been reported (Burklew et al., 2012). However, to our knowledge, there are no reports about Alt-responsive miRNAs from nodules of SNF legumes.

In this work we aimed to characterize the response of SNF common bean plants, inoculated with *Rhizobium tropici*, growing in low pH with excess Al and to delineate root and nodule Alt-responsive miRNAs. For comparison we also analyzed phenotypic Alt responses of nitrate-fertilized plants. A miRNA expression profile, based in hybridization of a miRNA macroarray (Valdés-López et al., 2010), was performed to identify Alt-responsive miRNAs in common bean nodules and roots. Expression analysis, based on real-time quantitative reverse transcriptase-PCR (qRT-PCR), was performed for selected Alt-responsive miRNAs and their predicted/validated target mRNAs. Proposed roles of the analyzed miRNA/target nodes in signaling pathways of the nodules/roots from common bean exposed to acidity/Alt are discussed in view of previous studies from other plant species subjected to similar stress. Our work contributes to increase the knowledge about Alt-responsive miRNAs in an agronomical important legume, extensively grown in acidic soils.

## Materials and Methods

### Plant Material and Growth Conditions

The common bean (*P. vulgaris* L.) Mesoamerican cv. Negro Jamapa 81 was used in this study. Seeds were surface sterilized in 70% (v/v) ethanol for 1 min followed by 10% (v/v) commercial sodium hypochlorite for 10 min and finally rinsed 5–6 times in sterile distilled water where they remained soaking for 12 h. Subsequently seeds were germinated on moist sterile paper towels in the dark at 30°C for 2 days. Germinated seedlings were grown in hydroponic system under controlled environmental conditions as previously described (Valdés-López et al., 2010). The hydroponic trays contained the nutrient solution reported by Franco and Munns (1982b). To induce Alt the pH of the nutrient solution was adjusted to 4.5 using 1 N HCl and it was supplemented with 70 μM AlCl_3_. For the control treatment, full-nutrient solution without excess Al, the pH was also adjusted to 4.5. Throughout every experiment the pH and volume of the nutrient solution from the hydroponic trays were controlled daily and the nutrient solution was changed every 3–4 days for fresh solution (with or without excess Al).

The AlCl_3_ concentration (70 μM) used in this work for Alt treatment was selected based in the results of the phenotypic analysis performed in SNF common bean plants subjected to 50, 70, or 100 μM AlCl_3_ as compared to control treatment. For this experiment common bean plants were grown under symbiotic conditions, as described next; these were harvested for phenotypic analysis 7 days after exposure to a different AlCl_3_ concentration.

For symbiotic condition, plantlets adapted to grow for 3 days in hydroponic trays with N-free Franco/Munns solution were inoculated with 5 ml of a saturated (over-night) liquid culture of *R. tropici* CIAT 899 (Graham et al., 1994). For Alt stress treatment the nutrient solution was changed at 12 days post-inoculation (dpi), when inoculated plants had already formed nodules, for a solution supplemented with 70 μM AlCl_3_. These plants were harvested 24 h (13 dpi) or 7 days (19 dpi) after Alt exposure. For control treatment fresh nutrient N-free Franco/Munns nutrient solution was changed at 12 dpi and inoculated plants were harvested at 13 or 19 dpi.

For non-symbiotic condition, full nutrient Franco/Munns solution was used. For Alt-stress treatment, 12 days after planting the nutrient solution was changed for one supplemented with 70 μM AlCl_3_ and plants were harvested 24 h or 7 days after Alt exposure. For control treatment, fresh nutrient solution with the same composition was changed after 12 days and plants were harvested at 13 or 19 days after planting.

In each experiment the expression of an Al-activated malate transporter (*ALMT*1, Phvul.001G081000^1^) marker gene for Alt (Chandran et al., 2008) was determined by qRT-PCR. In addition, visible morphological changes in roots and nodules from Alt plants as compared to control were checked in every experiment. The decrease in root length, pale pink or whitish colored nodules with a roughened external surface as well as increased expression of the *ALMT1* marker gene indicated the stress-nature of the treatment used.

Both control and stress (symbiotic or fertilized) treatments consisted of three independent plastic trays, with eight seedlings per tray. Three different sets of plants were considered for analysis. From the total plants in each experiment (24) a different number of harvested plants were used for each phenotypic, biochemical or molecular analysis as will be described below.

### Phenotypic Analysis

From the total number of plants in each experiment, 10 plants from each treatment (inoculated or fertilized) were harvested at each of the indicated time points for root length and dry weight (DW) determination. The length of the primary root was measured from freshly harvested plants; roots were cut and rinsed with tap water. After eliminating the excess of water with paper towels, each root was placed in a flat surface and the primary root was extended completely to measure it with a ruler. This procedure was done carefully to avoid breakage of the roots. Subsequently each root was dried in an oven at 60°C for 3 days and then weighed on an analytical scale to calculate the root DW. In experiments with inoculated plants, nodules were excised from the root before drying the roots. Student’s *t*-test was used to analyze the difference in root length and DW between control and Alt stressed plants.

Nitrogenase activity was determined by the acetylene reduction assay (Hardy et al., 1968) in detached nodulated roots from 10 plants form each treatment. Root samples with mature nodules were placed into 20 ml vials, 2 ml of acetylene were injected into the vial to create a 10% acetylene atmosphere. The vials were incubated at room temperature 30 min, 2 ml of the gas of each vial were removed and injected in the gas chromatographer to analyze ethylene concentration. Specific activity is expressed as nmol ethylene h^-1^ g^-1^ nodule DW. Student’s *t*-test was used to analyze the difference in nitrogenase activity of Alt stressed plants as compared to control plants.

For the phenotypic analyses described below, the microscopes used were: a light microscope SteREO (Zeiss) or a fluorescence optical microscope Axioskop 2 (Zeiss). From the total number of plants in each experiment, eight individual roots or nodules from each treatment were examined in every staining protocol.

Aluminum accumulation was detected by morin staining following the protocol reported by Tice et al. (1992). Common bean root tips and nodules slices were washed with 5 mM ammonium acetate buffer (pH 5.0) for 20 min, followed by 1 h incubation in 100 mM morin dissolved in the same buffer and finally washed with buffer for 20 min. Green fluorescence from Al-morin complexes was observed at 420 nm excitation and 510 nm emission wavelengths. Production of H_2_O_2_ ROS was detected after roots incubation for 35 min in a solution containing 200 μM CaCl_2_ plus 10 μM 6-carboxy-2′, 7′-dichlorodihydrofluorescein diacetate, di(acetoxymethyl ester) (DCF-DA) that emits fluorescence when interacting with H_2_O_2_ (Jones et al., 2006). ROS-fluorescence was observed at 488 nm excitation and 530 nm emission wavelengths. Lipid peroxidation in excised root and nodules was visualized after staining with Schiff’s reagent for 35 min, as reported by Yamamoto et al. (2001). Cell death, assessed by the loss of membrane integrity, was visualized by staining with Evans blue (Yamamoto et al., 2001) during 15 min for roots and 20 min for nodules. Accumulation of callose was determined as reported by Millet et al. (2010) using aniline blue. For this assay entire roots and nodule slices were used. Callose fluorescence was observed immediately under UV (390 nm excitation and 460 nm emission).

### Preparation, Hybridization and Data Analysis of miRNA Macroarrays

The approach used to identify miRNAs from nodules of common bean plants from control or Alt treatment was based on the hybridization of miRNA macroarrays as previously reported by Valdés-López et al. (2010). The steps of this protocol are briefly explained next.

Total RNA was isolated from 1 to 2 g frozen nodules of control or Al-treated common bean plants using LiCl precipitation method as reported previously (Valdés-López et al., 2008). Total nodule RNA samples were enriched for miRNAs by using flashPAGE fractionator (Ambion). These samples (hereafter termed ‘miRNA samples’) were preserved at -80°C until used for miRNA macroarray hybridization.

Forty-two synthetic DNA oligonucleotides (18–24 nts) corresponding to reverse complementary sequences of 42 mature miRNAs families were synthesized. Twenty-three of these DNA oligonucleotides corresponded to conserved miRNAs, 7 to miRNAs from soybean, 9 to miRNAs from common bean, and 3 to miRNAs expressed in *M. truncatula* nodules (Lelandais-Brière et al., 2009). The recently released *P. vulgaris* genome sequence (Schmutz et al., 2014^2^) was analyzed to verify that the precursors of each of the 42-selected miRNA were indeed encoded in the genome. Other DNA oligonucleotide probes complementary to different miRNA families that were used in the miRNA macroarray analysis reported by Valdés-López et al. (2010) were excluded from this analysis because these could not be mapped in the common bean genome. The sequences of the DNA oligonucleotide probes printed in the miRNA macroarray used in this work are provided in Supplementary Table S1. For miRNA macroarray preparation each DNA oligonucleotide probe was manually spotted on 2 cm × 3 cm Amersham Hybond-N^+^ membranes, dried at room temperature and UV cross-linked three times.

After hybridization with radiolabeled miRNA samples the macroarray membranes were washed, exposed to a Phosphor Screen System and scanned. Three independent miRNA macroarrays were hybridized with miRNAs isolated from three different plants (biological replicates) of each treatment. The signal intensity of each spot of the miRNA macroarrays was determined using ImageQuant 5.2 software (Molecular Dynamics, Sunnyvale, CA, USA). The signal intensity data were normalized with the average of the signal intensity of the printed miR159 that showed no significant variation across all the conditions tested and therefore has been used by our group in previous works (Naya et al., 2014; Nova-Franco et al., 2015). The normalized data were then used to analyze the level of expression of each miRNA in the nodules from plants grown in control and in metal-toxicity conditions. The raw and normalized signal intensity data from each miRNA macroarray hybridization experiment are shown in Supplementary Table S2.

For analysis of the differential expression of miRNAs in nodules from metal toxicity-stressed plants, the average normalized expression ratios (stressed:control) were obtained and subjected to Student’s *t*-test (*p* ≤ 0.05).

### Semi-quantitative and Real-time Quantitative Reverse Transcriptase-PCR (qRT-PCR) Analysis

Transcript level of the *ALMT1* marker gene in roots under control or Alt stress were analyzed by semi-quantitative RT-PCR that was performed by two-step RT-PCR using polythymine deoxynucleotide primer following the manufacturer instructions (Clontech Laboratories, Inc. Mount View, CA, USA). Annealing temperature was 55°C, 28 cycles were used. Primer oligonucleotide sequences are shown in Supplementary Table S3. Amplified RT-PCR products were resolved on 2% (w/v) agarose gels in Tris-acetate-EDTA buffer. The ubiquitin gene (*UBQ*) was included as a control for uniform RT-PCR conditions.

For the quantification of the transcript levels of mature miRNAs, cDNA was synthesized from 500 ng of total RNA the NCode miRNA First-Strand cDNA Synthesis (Invitrogen). Resulting cDNAs were then diluted and used to perform qRT-PCR assays using the Maxima SYBR Green/Fluorescein qPCR master mix (Fermentas, Hanover, MD, USA), following the manufacturer instructions. The transcript levels of selected common bean miRNA target genes were quantified by the one-step assay using the iScript One-Step RT-PCR Kit with SYBR Green (Bio-Rad, Hercules, CA, USA). Each qRT-PCR reaction contained 100 ng of RNA template, previously treated with DNase (Qiagen, Hilden, Germany). Both for mature miRNAs and for target genes transcript level determinations, qPCR reactions were run in a 96-well format with the iQ5 Real-Time PCR Detection System and iQ5 Optical System Software (Bio-Rad) with settings of 10 min at 50°C (cDNA synthesis), 5 min at 95°C (iScript reverse transcriptase inactivation), followed by 40 cycles for PCR cycling and detection of 30 s at 55°C. Supplementary Table S3 provides the sequences of the oligonucleotide primers used for qRT-PCR amplification of each gene.

Three biological replicates with two technical replicates each were carried out for the determination of transcript level of each gene or miRNA, RNA was extracted from differing sets of plants grown under similar treatment (control or Alt). Relative transcript levels for each sample were obtained using the ‘comparative *C*_t_ method’. The threshold cycle (*C*_t_) value obtained after each reaction was normalized to the *C*_t_ value of miR159 for miRNA levels or to the *C*_t_ value of the elongation factor 1 (EF1) gene (Phvul.004G060000) for target gene levels; these reference genes were constant across the conditions (Supplementary Table S2). The relative expression level was obtained by calculating the ΔΔ*C*_t_ values for the stress conditions used and the normalized *C*_t_ value (Δ*C*_t_) for the controls. The normalized fold expression levels were subjected to Student’s *t*-test (*p* ≤ 0.05).

## Results

### Response of Common Bean Plants to Al Toxicity

The objective of this work was to characterize the response of SNF common bean plants to acidic/Al-toxicity stress, aiming to describe the symptoms present as well as the miRNAs differentially expressed in active nodules from plants exposed to Alt, something that is yet poorly documented for this or other legumes. To achieve our objective, our experimental design took into consideration previous knowledge about the negative effect of low pH on rhizobia root colonization/infection and nodule development/function in common bean (Franco and Munns, 1982a; Vassileva et al., 1997). Such negative effects are related to the acid sensitivity of free-living rhizobia (Graham et al., 1994; O’Hara and Glen, 1994). Our experimental design, based on that reported several years ago by Franco and Munns (1982a), has been used by our group to describe the miRNA expression profile from previously developed nodules of SNF common bean plants that were exposed to a nutrient deficiency, acidity or Mn toxicity. In short, plantlets adapted to grow in hydroponic conditions were inoculated with *R. tropici* CIAT 899 that is acid tolerant (Graham et al., 1994), when functional nodules were formed stress was imposed by changing the nutrient solution to one containing 70 μM AlCl_3_. For the control treatment, another set of nodulated plants continued to grow in N-free nutrient solution. For both treatments the pH of the solution was adjusted to 4.5 and was controlled throughout the experiment, this allowed to separate the effect of Al from that of low pH.

**FIGURE 1:**
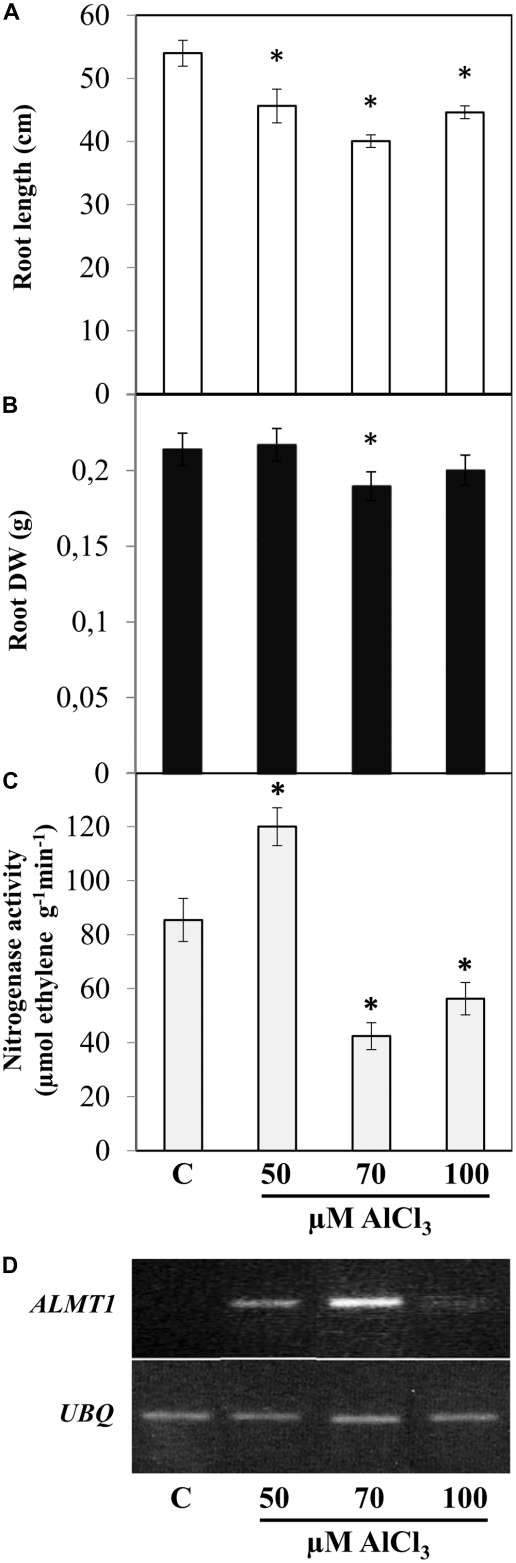
**Effect of different AlCl_3_ concentrations on symbiotic nitrogen-fixing common bean plants.** The indicated AlCl_3_ concentration was added to (12 dpi) *Rhizobium tropici*-inoculated plants with active nodules for 7 days. C: control condition without AlCl_3_. Values of root length **(A)**, root dry weight (DW) **(B)** or nitrogenase specific activity **(C)**, as determined by the acetylene reduction assay, are mean ± SE from 10 different plants (biological replicates) per treatment. Student’s *t*-test was used to analyze the difference in each parameter as compared to control treatments. Columns marked with star (^∗^) represent significantly different means according to the statistical analysis (*p* ≤ 0.05). **(D)** The expression of the Alt-stress marker gene *ALMT1* was evaluated by semi-quantitative RT-PCR from roots of plants inoculated plants grown in each treatment, as indicated. The ubiquitin gene (*UBQ*) was included as a control for uniform RT-PCR conditions. Shown is a representative gel from a total of three experiments.

The degree of toxic effect induced by a certain Al concentration varies considerably depending on the plant species/genotype, the experimental conditions and other factors (Kochian et al., 2005). Therefore initially we tested different AlCl_3_ concentration in order to select the adequate treatment for SNF common bean plants to be used this work. After 12 dpi *R. tropici*-nodulated common bean plants were transferred to nutrient solution supplemented with 50, 70, or 100 μM AlCl_3_ (pH 4.5) and were harvested 7 days after Al exposure for phenotypic analysis (**Figure 1**). As compared to control condition (N-free nutrient solution, pH 4.5), SNF common bean plants exposed to 70 μM AlCl_3_ showed highest reduction in root length (30%, **Figure 1A**) and in root DW (14%, **Figure 1B**) as compared to plants in the other Al-stress treatments tested. In addition, the nitrogenase activity (acetylene reduction assay) from nodules under 70 μM AlCl_3_ was the lowest (50%, **Figure 1C**). These data are in agreement with the expression level of *ALMT*1 marker gene observed in roots of plants from the different treatments; it was highest in roots from plants under 70 μM AlCl_3_ treatment (**Figure 1D**). On this basis, for this work the selected AlCl_3_ concentration for Alt in common bean was 70 μM.

**FIGURE 2:**
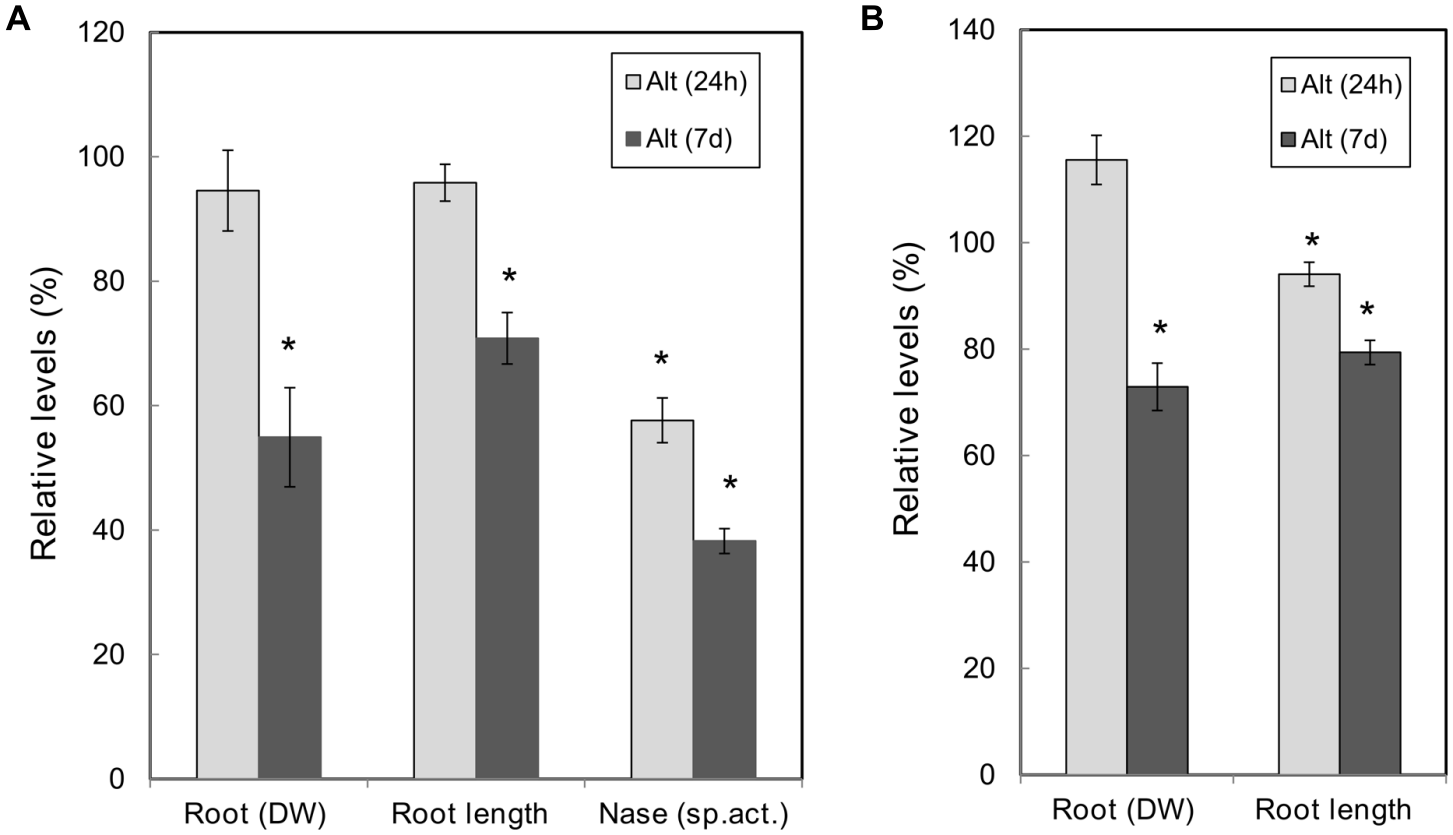
**Effect of Al toxicity on common bean plants.** High Al (70 μM AlCl_3_) was added to (12 dpi) *R. tropici*-inoculated plants with active nodules **(A)** or to (12 days) fertilized plants **(B)**, for 24 h or 7 days as indicated. Values are expressed relative to those from nodulated **(A)** or fertilized **(B)** plants grown in control condition (100%). Nitrogenase specific activity, as determined by the acetylene reduction assay, is expressed per nodule DW. Values are the mean ± SE for ten biological replicates from different plant sets. Student’s *t*-test was used to analyze the difference in each parameter between plants grown under stress vs. control treatments. Columns marked with star (^∗^) represent significantly different means according to the statistical analysis (*p* ≤ 0.05).

Due to the genotypic variability and diverse experimental conditions it is difficult to reach a consensus on the timing for Alt in plants; different responses have been observed at early or at late exposure to Al-stress (Kochian et al., 2005). In this work we analyzed common bean response to Alt (70 μM) at two periods of Al exposure. The long period selected for Alt-response analysis was 7 days, based in our previous work (Valdés-López et al., 2010) and we included 24 h exposure for analysis of an earlier response. **Figure 2A** shows the data of the phenotypic analysis performed in SNF bean plants exposed to Alt treatment (24 h or 7 days) as compared to control plants. No significant change was observed in the leaf area or the nodule DW from SNF plants under Alt treatments (data not shown). SNF plants exposed for 7 days to Alt showed a decreased in root DW (50%) and root length (20%), while plants from 24 h treatment did not show changes in these parameters. However, as evidenced by nitrogenase activity (acetylene reduction assay) values, both Alt treatments affected nodule function since a 43 and 62% reduction at 24 h and 7 days, respectively, was observed.

We compared the phenotypic analysis of SNF common bean plants with full-nutrient fertilized common bean plants subjected to Alt for short or long periods. Similar condition as those for nodulated plants were used, Alt was added for 24 h or 7 days to roots of plants pre-grown for 12 days in full-nutrient solution. No alteration in leaf area was observed in all the treatments analyzed (data not shown). In Al-treated fertilized plants a decrease in root DW (28%) was observed only at 7 days treatment, while a decrease in root length was observed both at 24 h (6%) and 7 days (25%) treatments (**Figure 2B**).

**FIGURE 3:**
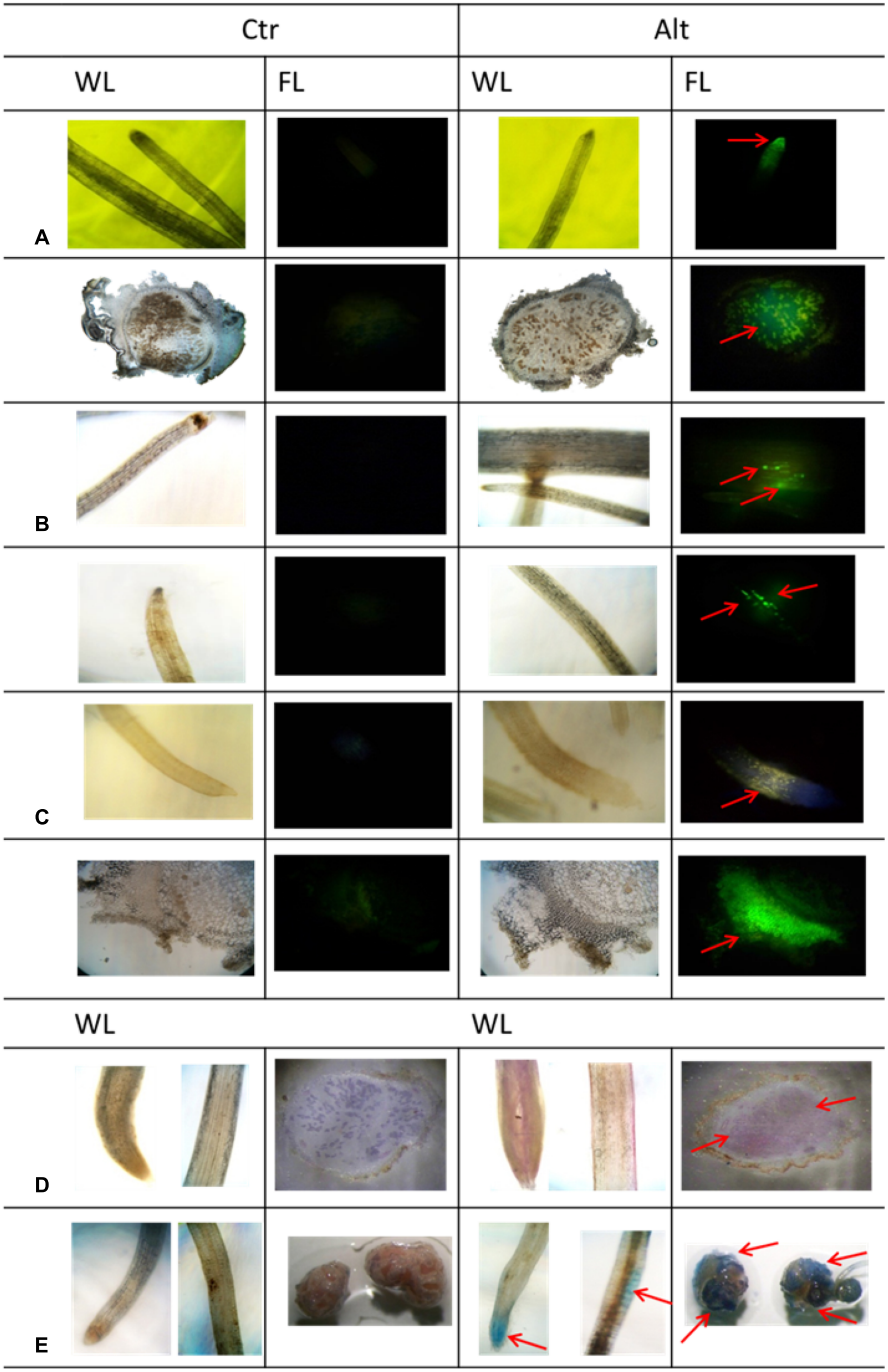
**Histological analysis of Al toxicity response in roots and mature nodules of *R. tropici*-inoculated common bean plants.** SNF common bean plants were grown in control (Ctr) or in Al- toxicity (Alt) conditions. **(A)** Accumulation of Al evidenced by Al-morin fluorescence. Magnification = 10X, for roots and nodules **(B)** ROS (H_2_O_2_) production detected by DCF-DA fluorescence. Magnification = 10X **(C)** Callose distribution evidenced by fluorescence emitted after aniline blue staining. Magnification = 10X for roots and 20X for nodules. Magnification = 10X for roots and 8X for nodules **(D)** Lipoperoxidation visualized after Schiff’s reagent staining **(E)** Cell death visualized after Evans blue staining. Magnification = 10X for roots and 4X for nodules. Red arrows point out the specific symptoms commented in the text. WL, white light, FL, fluorescent light. Each histological analysis was repeated in eight roots or nodule samples from different plants grown in control or Alt treatment, representative images are shown.

Following the characterization of the response of SNF common bean to Alt, we performed a histological analysis, in both roots and nodules, to observe symptoms known to be specific and characteristic of roots from different plants exposed to excess Al (Kochian et al., 2005; Vitorello et al., 2005). This was done after long period (7 days) exposure to Alt because this treatment resulted in the highest effect on SNF plants (**Figure 2A**). Fluorescence resulting from accumulation of Al, after morin staining, was observed in the root tips and in the infection zone of mature nodules of plants exposed to Alt, while no fluorescence was observed in organs from control plants (**Figure 3A**). ROS (H_2_O_2_) accumulation, evidenced as fluorescence after DCF-DA treatment, was high in the Alt roots elongation zone as compared to roots from control plants (**Figure 3B**). This test was not useful to detect ROS from nodules since DCF-DA could not penetrate into the nodules and no fluorescence could be observed. Callose-fluorescence was observed in the elongation zone of bean roots (**Figure 3C**). In nodules, the callose-fluorescence was observed mainly on the outer layer (**Figure 3C**). Peroxidative damage of membrane lipids (lipoperoxidation) due to the stress-related increase in high toxic ROS is often associated with Alt (Cakmak and Horst, 1991). The Schiff’s reagent was used to visualize aldehydes derived from lipoperoxidation. In roots from Alt bean plants an intense pink staining was observed mainly in the elongation zone and in the whole infection zone of stressed nodules (**Figure 3D**). An oxidative damage leads to a disturbance of cellular homeostasis that could result in cell death thus we used Evan’s blue staining to assess cell death in Alt roots and nodules. Cell death was evident in the tips and the elongation zone of roots of stressed bean plants (**Figure 3E**). In Al-stressed nodules we observed a build up of tissue that gave a roughened texture to the external surface. Evan’s staining of entire nodules revealed that cell death could be occurring in the rough external layer (**Figure 3E**).

### miRNAs Expression Profile in Nodules of Common Bean Plants Under Alt

Recent studies have identified miRNAs that are differentially expressed in tissues of plants exposed to Alt; to our knowledge, these do not include legume nodules (Zhou et al., 2008; Lima et al., 2011; Burklew et al., 2012; Chen et al., 2012; Zeng et al., 2012). Alt-responsive miRNAs are likely to play important roles in the regulation of plant response/adaptation to this stress. In this work we aimed to identify miRNAs from common bean nodules exposed to high Alt for 7 days; in this treatment SNF bean plants showed major phenotypic alterations (**Figure 2**).

The miRNAs expression profile analysis from Alt nodules was performed through hybridization of a miRNA macroarray; the membranes used contained 42 DNA oligonucleotide probes (Supplementary Table S1) complementary to genome-mapped miRNAs that are expressed in different tissues of common bean plants (Peláez et al., 2012).

The data on normalized expression level (stressed:control) of each of the 42 miRNA families analyzed through macroarray hybridization revealed that 28 miRNAs were differentially regulated in Alt-stressed common bean nodules; half of these were up-regulated (**Table 1**). Among the up-regulated miRNAs we found 10 conserved miRNAS, from these miR164 and miR396 showed the highest response; while eight conserved miRNAs were down-regulated (**Table 1**). Also we found 10 Alt-responsive miRNAs that have been identified in one or more legume species (common bean, soybean and/or *M. trucatula*), thus putative legume-specific miRNA families, six of these were down-regulated (**Table 1**).

**Table 1:** MicroRNA (miRNA) expression in nodules of common bean plants grown under control or Alt condition.

miRNA	Expression Ratio (±SE)	*P*-value
miR 160	1.91 (±0.01)	3.60E-05
miR 164	5.35 (±0.03)	8.06E-06
miR 165	1.64 (±0.02)	0.00035
miR 166	1.94 (±0.06)	0.0002
miR 170	5.70 (±0.52)	0.00104
miR 172	1.63 (±0.10)	0.00216
miR 390	2.57 (±0.01)	3.38E-06
miR 393	2.12 (±0.08)	0.00464
miR 395	2.02 (±0.01)	4.42E-05
miR 396	5.98 (±0.12)	0.00156
pvu-miR159.2	3.88 (±0.17)	0.0001
pvu-miR1509	2.13 (±0.01)	0.00088
pvu-miR1511	2.89 (±0.01)	9.53E-06
pvu-miR 2118	2.21 (±0.01)	7.95E-05
miR 156	-1.16 (±0.009)	0.00387
miR 157	-3.57 (±0.004)	1.57E-05
miR 167	-1.38 (±0.05)	0.04902
miR 169	-10.0 (±0.003)	8.10E-06
miR 319	-2.30 (±0.01)	0.00022
miR 398	-1.45 (±0.01)	0.04504
miR 399	-2.56 (±0.03)	0.00120
miR 408	-1.29 (±0.008)	0.02278
pvu-miR1514a	-1.66 (±0.001)	0.00515
pvu-miR1515	-3.70 (±0.001)	0.01827
pvu-miR2119	-2.63 (±0.03)	0.01199
gma-miR1521	-2.00 (±0.006)	7.00E-05
gma-miR1534	-2.56 (±0.005)	0.00415
mtr-miR2586	-1.60 (±0.005)	0.015564

*High aluminum was added at 12 dpi for 7 days. The significant expression ratios (Alt:control) are the average of normalized signal intensity values from three biological replicates of miRNA-macroarrays. Names of non-conserved miRNAs, reported previously (Arenas-Huertero et al., 2009; Lelandais-Brière et al., 2009; Valdés-López et al., 2010), include abbreviation of the legume species where these were first annotated: pvu, *Phaseolus vulgaris*, gma, *Glycine max*, and mtr, *Medicago truncatula*. For ratios lower than 1 (miRNAs down-regulated in stress), the inverse of the ratio was estimated and the sign was changed.*

### Expression Analysis of Selected Metal Alt-Responsive miRNAs and their Target Genes

**FIGURE 4:**
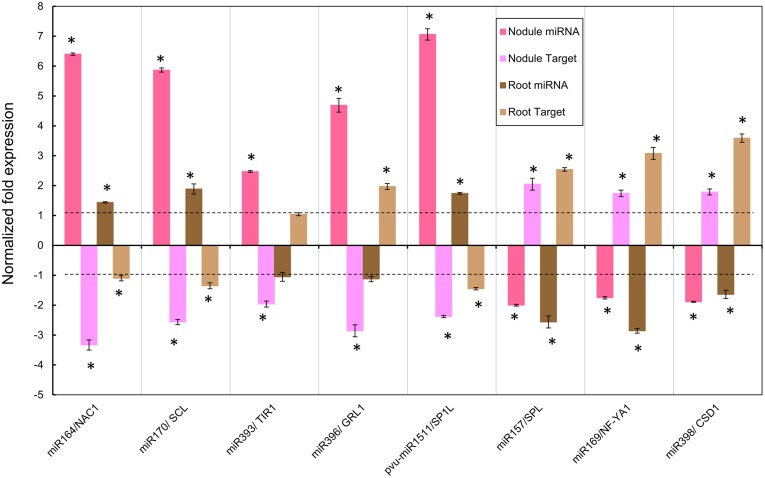
**Normalized fold expression levels of selected miRNA/target mRNA nodes in fertilized roots and in SNF nodules of common bean plants under Alt for long period.** High Al was added to (12 dpi) *R. tropici*-inoculated plants with active young nodules or to (12 days) fertilized plants for 7 days before harvesting. miRNAs and target genes expression levels were determined by qRT-PCR in nodules of inoculated plants or in roots of fertilized plants. Values represent the normalized expression ratios (stress:control) given as the average of three biological replicates. For ratios lower than 1, the inverse of the ratio was estimated and the sign was changed. Columns marked with star (^∗^) represent significantly different means according to the statistical analysis (*p* ≤ 0.05). NAC1, NAM/ATAF/CUC transcription factor; SCL, SCARECROW-like protein; TIR1, TRANSPORT INHIBITOR RESPONSE 1-like protein; GRL1, GROWTH-REGULATING FACTOR 1; SP1L, SPIRAL-like protein 1; SPL, SQUAMOSA PROMOTER-BINDING protein-like; NF-YA1, NUCLEAR FACTOR YA1; CSD1, Cu/Zn SUPEROXIDE DISMUTASE.

**FIGURE 5:**
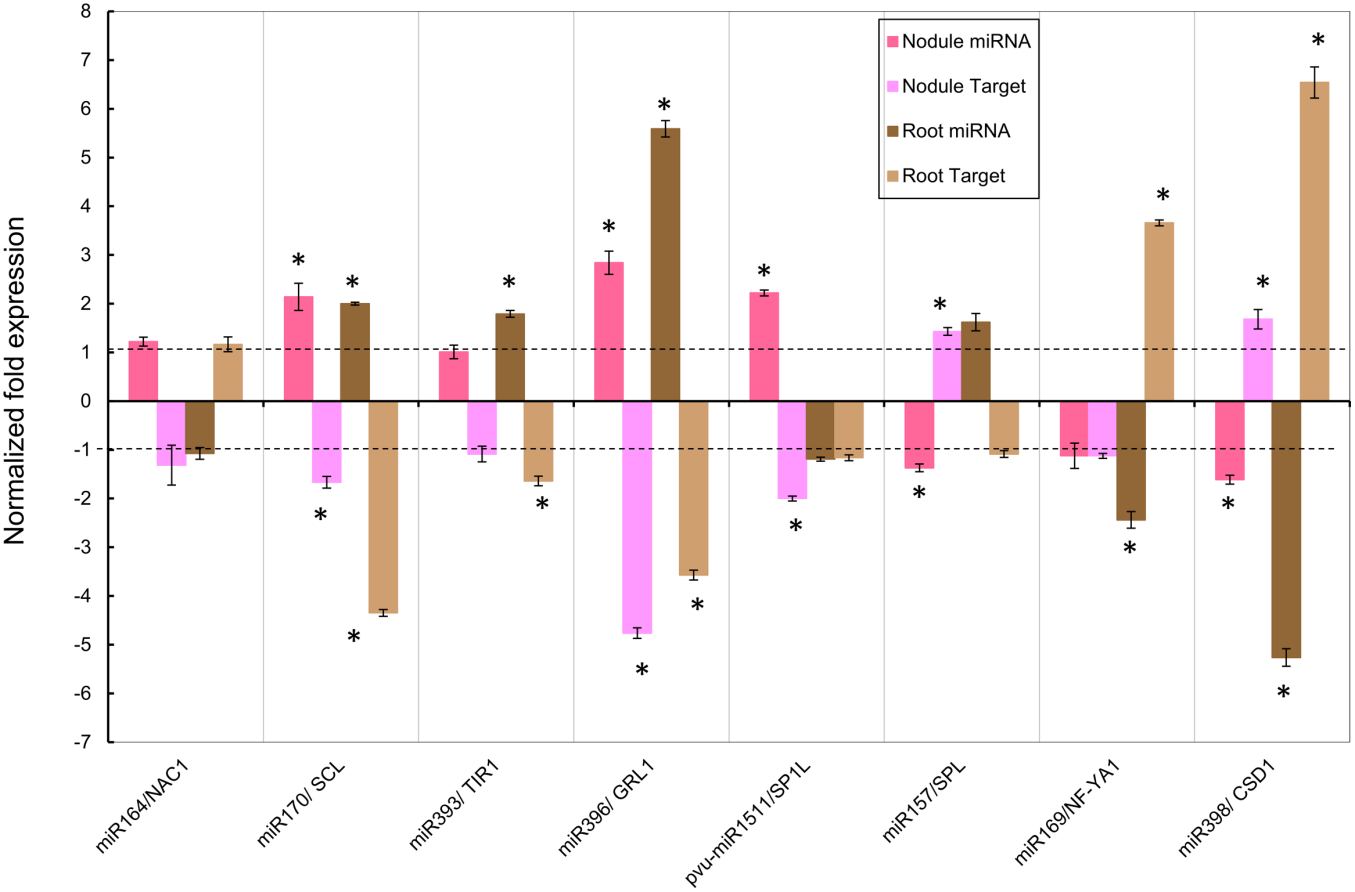
**Normalized fold expression levels of selected miRNAs/target mRNA nodes in fertilized roots and in SNF nodules of common bean plants under Alt for short period.** High Al was added to (12 dpi) *R. tropici*-inoculated plants with active young nodules or to (12 days) fertilized plants for 24 h before harvesting. miRNAs and target genes expression levels were determined by qRT-PCR in nodules of inoculated plants or in roots of fertilized plants. Values represent the normalized expression ratios (stress:control) given as the average of three biological replicates. For ratios lower than 1, the inverse of the ratio was estimated and the sign was changed. Columns marked with star (^∗^) represent significantly different means according to the statistical analysis (*p* ≤ 0.05). See **Figure 4** for target genes description.

We selected eight Alt-responsive miRNAs from common bean nodules, including both up-regulated and down-regulated examples, to validate the expression rates obtained in the miRNA macroarray experiment (**Table 1**) using the alternative method of qRT-PCR. Besides validating the expression of selected miRNAs on nodules from SNF plants exposed for long period to Al (**Table 1**) we extended the analysis to nodules under Alt-stress for short period, and also to roots from fertilized plants exposed to Al for long or short periods (**Figures 4** and **5**).

To gain insight into specific roles of miRNAs in common bean response to Al stress, we included a qRT-PCR expression analysis of the genes targeted by the selected miRNAs (**Figures 4** and **5**). Very few common bean miRNAs target genes have been experimentally validated (Arenas-Huertero et al., 2009); we considered this an important issue for the selection of miRNAs/target nodes to be used for expression analysis in this work. So six, out of eight, miRNAs that were selected for expression analysis in this work have a validated target gene in common bean (Arenas-Huertero et al., 2009); these include four up-regulated and two down-regulated miRNAs according to data from macroarrays (**Table 1**). Each miRNA may target several genes, from the same or from different gene families (Kraiwesh et al., 2012; Sunkar et al., 2012; Rogers and Chen, 2013). In order to define this for common bean miRNAs, further genome-wide analysis based in the recently published genome sequence (Schmutz et al., 2014) as well as experimental validation for predicted targets is required. However, in this work we focused in the expression analysis of the only validated (or predicted) target gene for each selected miRNA (Arenas-Huertero et al., 2009; Valdés-López et al., 2010). The selected up-regulated miRNAs and their corresponding validated target genes (Arenas-Huertero et al., 2009) are: miR164/NAC1 (NAM/ATAF/CUC transcription factor), miR170/SCL (SCARECROW-like protein transcription factor), miR393/TIR1 (TRANSPORT INHIBITOR RESPONSE 1-like protein) and miR396/GRL1 (GROWTH-REGULATING FACTOR-like protein). The selected down-regulated miRNAs and their corresponding validated target (Arenas-Huertero et al., 2009; Naya et al., 2014) are: miR157/SPL (SQUAMOSA PROMOTER-BINDING PROTEIN-LIKE) and miR398/CSD1 (Cu/Zn SUPEROXIDE DISMUTASE). We also selected miR169 that showed the highest value for down-regulation in Alt-stressed common bean nodules (**Table 1**). This was the first miRNA whose role in nodule development was demonstrated in *M. truncatula*; it targets the transcription factor NF-YA1 (NUCLEAR FACTOR YA1, previously called HAP2) was experimentally validated in *M. truncatula* and in soybean and it was predicted by bioinformatics analysis for common bean (Combier et al., 2006; Arenas-Huertero et al., 2009; Song et al., 2011). In addition, we selected pvu-miR1511, up-regulated in Alt nodules (**Table 1**), its predicted target in common bean target gene is SP1L (SPIRAL-like protein, a microtubule-associated protein; Arenas-Huertero et al., 2009); this miRNA has been found in the legumes *M. truncatula* and soybean (Lelandais-Brière et al., 2009; Song et al., 2011; Formey et al., 2014) but its regulatory function remains unknown.

**Figure 4** shows the data of the expression ratios of the selected miRNAs and their target genes in active nodules that were exposed 7 days to Alt. The tendency (up- or down-regulation) in the response to Alt of each miRNA tested using qRT-PCR approach was similar to that shown **Table 1**, thus validating the miRNA macroarray results. However, there was variation in the expression ratio values obtained from macroarrays as compared to those from qRT-PCR analysis (**Figure 4**, **Table 1**) that could be attributable to different sensitivities of the two methods. In every case, the expression ratio of the target gene showed the expected inverse correlation with that of its miRNA (**Figure 4**). The respective target genes of the miRNAs miR164, miR170, miR393, miR396, and pvu-miR1511 were down-regulated pointing to the miRNA-induced target cleavage (**Figure 4**).

We also analyzed the expression of the selected miRNA/target nodes in developed roots from fertilized plants subjected to Alt for 7 days (**Figure 4**). As shown in **Figure 4**, miR164, miR170, and miR1511 were up-regulated while miR157, miR169, and miR398 were down-regulated in 7 days Alt roots. The target genes of these Alt root responsive miRNAs showed an inverse correlation with that of their miRNA (**Figure 4**). miR393 and miR396 did not show a significant response in Alt roots (**Figure 4**).

To assess if the observed miRNA response was characteristic of a long-time exposure to Alt (**Figure 4**) we compared it to that of short Alt exposure thus determining the expression of the selected miRNA/target nodes in nodules and roots exposed to high Al for 24 h (**Figure 5**). In 24 h Alt nodules miR170, miR396, and pvu-miR1511 were significantly up-regulated, miR157 and miR398 were significantly down-regulated and their target genes showed the respective inverse correlation; whereas miR164, miR393, and miR169 did not show a significant response (**Figure 5**). The expression analysis of selected miRNA/target nodes in 24 h Alt roots from fertilized plants revealed up-regulation of miR170, miR393, and miR396 and down-regulation of miR169 and miR398, with the corresponding inverse correlation of their target genes; whereas miR164, miR157, and pvu-miR1511 did not show a significant response (**Figure 5**)

## Discussion

In this work we report the negative effects of Alt to SNF and fertilized common bean plants, grown hydroponically in acidic nutrient solution supplemented with 70 μM AlCl_3_. This AlCl_3_ concentration is within the range of those found in acidic soils and those that have been previously used for Alt-stress experiments in common bean and in soybean (Franco and Munns, 1982a; Menzies et al., 1994; Rangel et al., 2007; Yang et al., 2009; Kopittke et al., 2015). In this work we used the black-seeded “Negro Jamapa 81” cultivar and *R. tropici* CIAT 899, an acid pH tolerant rhizobia strain (Graham et al., 1994), as inoculant. Though Franco and Munns (1982a) reported that black seed common bean varieties are less sensitive to acid soils with high Al concentration as compared to non-black seed varieties, Blair et al. (2009) reported that Mesoamerican common bean genotypes are less resistant to Al than Andean gene pools. The later report, aimed to identify Al resistant Andean common bean genotypes, analyzed 36 genotypes including 11 from the Mesoamerican gene pool but Negro Jamapa was not reported in this analysis (Blair et al., 2009). To our knowledge the degree of Al/acid soil resistance of the Negro Jamapa 81 cultivar remains to be analyzed, though this is out of the scope of our present work. The acid soils/Al stress resistance of both symbionts is likely to influence the survival/growth of common bean plants in the treatment used and also the miRNA response. Interestingly, future research may define if the Alt responsive common bean miRNAs identified account for general responses to the stress or if the response may vary among varieties with different adaptation/tolerance to Alt in acid soils.

A decrease in root length was the main and primary effect observed in common bean plants under Alt. After a short period under Alt common bean plants showed *ca.* 6% decrease in root length, equivalent to 2 cm shorter roots as compared to roots from control plants. This result is in agreement with previous works in common bean showing that the transition and the elongation zones of the root are the major targets of Al injury resulting in a rapid inhibition of root elongation (Rangel et al., 2007; Yang et al., 2009). After long period of Al exposure higher decrease in root length was observed, in inoculated and in fertilized plants. These results are in agreement with previous studies indicating that nodulated legumes are more sensitive to Al and Mn toxicity than plants fertilized with mineral N (Hungria and Vargas, 2000). Alt-stressed common bean nodules showed a decrease in nitrogenase activity together with accumulation of Al in their infected zone, thus indicating the presence of high Al in both symbionts. Bacteria under excess Al utilize Fe transport systems for Al uptake that interfere with their ability to capture Fe, an essential micronutrient required for rhizobial nitrogenase activity in rhizobia (Davis et al., 1971; Rogers et al., 2001). Species like *Sinorhizobium meliloti* and *Bradyrhizobium* growing *ex planta* are extremely sensitive to Al since it affects the enzymatic activities for nitrate and nitrite reduction, nitrogenase and uptake hydrogenase (Arora et al., 2010).

The Alt stressed bean roots and nodules showed characteristic Al-stress symptoms observed in roots from different plants such as accumulation of ROS and callose as well as lipoperoxidation. Noticeably, callose and H_2_O_2_ (ROS) accumulation co-localized in the bean root elongation zone similar as in Alt maize plants (Jones et al., 2006). Al-stressed maize root cells induce callose accumulation and cell wall/plasma membrane rigidification as well as an oxidative burst (ROS) with increasing cytoplasmic Ca^2+^ that leads to activation of the callose synthase enzyme (Jones et al., 2006). Though callose has been observed, in the cell walls of some yeasts, fungi and bacteria (Stone and Clarke, 1992) we did not observe callose-fluorescence in the nodule infection zone where *R. tropici* bacteroids reside, but in the external layers of Alt nodules. We propose that this may function as a protective barrier against Al uptake. It has been reported that callose accumulation can prevent higher uptake of Al and other metals (Van de Venter and Currier, 1977; Wissemeier and Horst, 1992) and also it may prevent pathogen infection (Kohler et al., 2002).

The identification of Alt-responsive miRNAs is a first step toward unraveling their regulatory role for plants adaptation/defense to this stress. To our knowledge, there is only one report about nutrient-deficiency and Mn-toxicity responsive miRNAs from common bean nodules (Valdés-López et al., 2010). In the present work we identified 28 Alt-responsive miRNAs in common bean nodules, using a miRNA macroarray hybridization approach proven to be inexpensive and suitable for this type of analysis (Valdés-López et al., 2010). However, in this work the miRNA macroarray design was improved by including only miRNAs encoded by and expressed from the common bean genome (Peláez et al., 2012; Schmutz et al., 2014), thus avoiding detecting non-specific hybridization signals that we now interpret as false positives.

**FIGURE 6:**
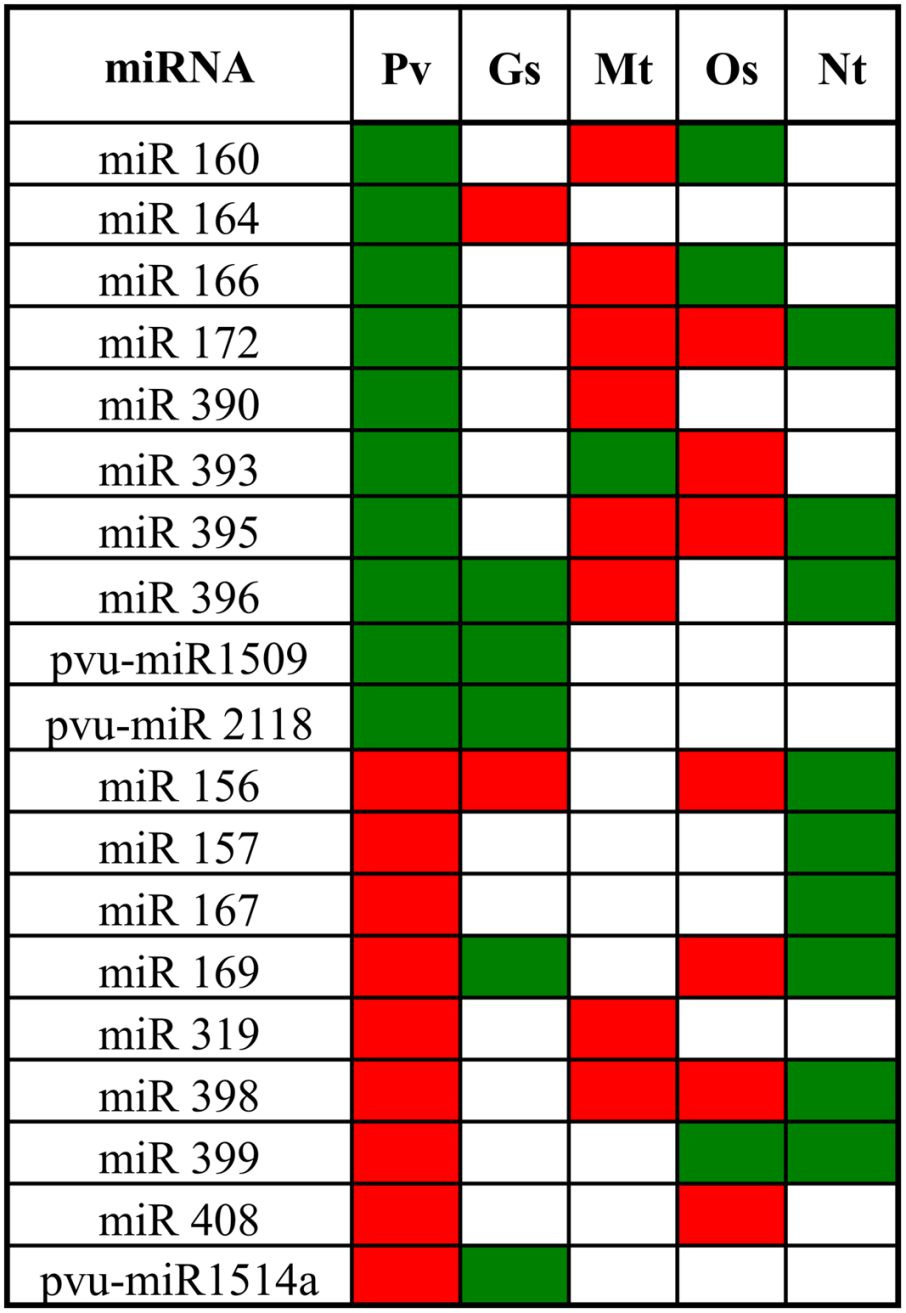
**Alt-responsive microRNAs from several plant species.** Pv, *Phaseolus vulgaris*, Gs, *Glycine soja*, Mt, *Medicago truncatula*, and Nt, *Nicotiana tabacum*. Green, up-regulation and red, down-regulation. References: Zhou et al. (2008), Lima et al. (2011), Burklew et al. (2012), Chen et al. (2012), and Zeng et al. (2012).

Expression profile of Al-stress responsive miRNAs has been reported for wild soybean, *M. truncatula*, rice and tobacco (Zhou et al., 2008; Lima et al., 2011; Burklew et al., 2012; Chen et al., 2012; Zeng et al., 2012; Yang and Chen, 2013; He et al., 2014). We did a comparative analysis of common bean miRNA families identified in this work with those found in other plant species subjected to similar Al-stress. As shown in **Figure 6**, 19 out of 28 miRNAs identified for common bean nodules are shared by one of more of the Al-stressed plant species; the rest miRNA families only identified in Alt common bean include 6 that have been found only in legumes (common bean, soybean and/or *M. truncatula*) and could represent family- or species-specific miRNAs (Song et al., 2011; Peláez et al., 2012; Formey et al., 2014). We identified three of these miRNA families (pvu-miR1509, pvu-miR2118, and pvu-mIR1514) only in Al-stressed common bean and wild soybean (*G. soja*; **Figure 6**), the latter is the ancestor of the domesticated soybean (*Glycine max*), a widely grown legume crop that is phylogenetically related to common bean (Zeng et al., 2012; Schmutz et al., 2014). *M. truncatula*, rice and tobacco shared 9, 10 and 9 Alt-responsive conserved miRNAs with common bean though several of these showed different trend (up- or down-regulation) as compared to that in common bean. It should be noted that Alt-responsive miRNAs were identified in different tissues: nodules for common bean, roots for wild soybean and rice, root tips for *M. truncatula* and seedling for tobacco (Lima et al., 2011; Burklew et al., 2012; Chen et al., 2012; Zeng et al., 2012). The commonalities among Alt-responsive miRNA families from different species indicate their role in the regulation of general relevant plant responses to this stress, while differences among the response on miRNA families may be related to specific roles in a certain plant tissue and/or species.

In this work we confirmed the macroarray results of eight selected Alt responsive miRNAs through the qRT-PCR expression analysis. This was complemented with the selected miRNA expression analysis in nodules at short period and roots at long and short periods under Alt. The action of miRNAs is exerted through the silencing of their corresponding target gene(s). To this end, we analyzed the expression level of the genes targeted by the selected Alt responsive nodule miRNAs. These showed the expected opposite trend to the miRNA expression, thus indicated the miRNA-induced target cleavage. The miRNA/target nodes analyzed in this work are known to be involved in relevant signaling pathways that regulate developmental processes or stress responses in different plants and thus allow us to propose their role in the response to Alt of common bean roots and nodules.

In general miRNA response in Al-stressed roots was more significant at short period, something that is in agreement with the roots sensing Alt effects in first place and showing earliest stress responses (Kochian et al., 2005; Vitorello et al., 2005). In this regard we observed that miR393 and miR396 did not respond in 7 days fertilized roots whereas these were up-regulated at short period, while miR169 and miR398 were down-regulated at a higher level in short- than in long-period stress. By contrast, generally a higher miRNA nodule response was observed at 7 days Alt, so perhaps miRNAs are have more important roles in trying to maintain nodule function even in prolonged Al-stress. Notably, nitrogenase activity was already considerably (43%) affected after 24 h Alt treatment so it seems that common bean nodule dysfunction precedes the turn-on of Alt stress signaling pathways related to miRNA differential expression. Although all the selected miRNA have a significant respond in 7 days Alt nodules, half of these -miR164, miR169, miR393, and pvu-miR1511- did not respond at short period of Al exposure.

Our analysis allows comparing miRNA responses to Al exposure in roots from fertilized plants vs. nodules from SNF plants. At long Al exposure, miR164, miR170 and miR1511 were highly up-regulated in nodules and showed the same trend in roots though to lower levels. Their corresponding target genes showed down-regulation thus indicating an important role for silencing NAC1 and SCL TF -involved auxin signaling and developmental processes, respectively- in both tissues under Alt. Similarly miR157, miR169, and miR398 were down-regulated in nodules and in roots with the corresponding up-regulation of their target genes (SPL and NF-YA1 TF and CDS1, respectively) that may have a relevant role in coping with Al stress in both organs through transcriptional regulation or ROS detoxification. By contrast at long Al exposure miR393 and miR396 showed a tendency for down-regulation in fertilized roots, although this was not statistically significant, while in nodules these miRNAs showed high up-regulation with the corresponding target gene silencing; thus indicating a relevant and more specific role in SNF Al stressed nodules.

We observed that miR164 and miR393 were highly up-regulated and their corresponding target genes NAC1 (transcription factor) and TIR1 (auxin receptor) were down-regulated in nodules exposed to Al for long period, while in 7 days fertilized roots miR164 showed a minor up-regulation and miR393 did not respond. miR164 was also reported as Alt-responsive in wild soybean and miR393 in *M. truncatula* and in rice (**Figure 6**). In *Arabidopsis*, the miR164/NAC1 and miR393/TIR1 nodes are involved in the auxin-signaling pathway that controls lateral root development (Guo et al., 2005; Navarro et al., 2006; Chen et al., 2011). In legumes the auxin/cytokinin ratio is strictly controlled and plays an important role in nodule development during the legume-rhizobia symbiosis (Ferguson and Mathesius, 2003). We propose that the auxin signaling pathway is a relevant component in the signal transduction for the response of common bean nodules to Al-stress. In addition, *Arabidopsis* miR393 and its target AFB3 (another auxin receptor) is a unique N-responsive node that regulates auxin response and controls root system architecture (lateral root formation) in response to N availability (Vidal et al., 2010). Though AFB3 has not been validated as miR393 target in legumes, we found AFB3 orthologs from soybean (Gmax19g27280) and from common bean (Phvul.001G087000) with putative miR393 binding sites within their coding region. Diminished nitrogenase activity in Alt common bean nodules would result in low N content, something that may regulate the expression of miR393 as observed.

Common bean roots and nodules under Alt for short and long periods showed up-regulation of miR170 and the corresponding down-regulation of its target SCL (transcription factor). The miR170/SCL node has been involved the gibberellin signaling pathway. Gibberellins promote cell elongation involved in root growth (Inada et al., 2000) and regulate lateral root formation through interactions with auxins and other hormones (Gou et al., 2010). In *Allium sativum* root tip cells the presence of gibberellins has been related with the restoration of lipid peroxidation and genotoxicity by metals such as cadmium (Celik et al., 2008). In nodules gibberellins biosynthesis is up-regulated during later stages of nodulation, these are required for proper mature nodule structure (Hayashi et al., 2014). We propose that the role of miR170/SCL and gibberellin signaling is relevant for the response of common bean roots/nodules to Al-stressed and it may be related to the regulation of characteristic responses such as root growth and lipid peroxidation. Notable, miR170 was not reported as Alt-responsive in wild soybean or *M. truncatula* roots (Chen et al., 2012; Zeng et al., 2012), thus indicating that its regulatory role is rather specific to mature nodule structure/function in common bean and maybe in other legumes.

We observed that miR398 was down-regulated and its target gene CSD was up-regulated in common bean roots and nodules under Alt for short and long periods. Similar response for miR398 was observed for *M. truncatula* and rice roots (**Figure 6**). miRNA398 was the first miRNA described as oxidative stress responsive in plants (Sunkar et al., 2006). Oxidative stress generated upon exposure to toxic concentrations of metals like copper (Cu), suppresses *Arabidopsis* miR398 expression that is essential for the accumulation of CSD1 and CSD2 required for detoxification of ROS (Sunkar et al., 2006). In common bean miR398/CSD1 node responds to oxidative stress and high ROS production resulting from Cu toxicity and from biotic interactions (Naya et al., 2014). It is known that Alt generates oxidative stress and ROS production in plants (Yamamoto et al., 2001; Kochian et al., 2005; Navascués et al., 2011), here we evidenced ROS increase and lipoperoxidation in Alt common bean nodules; therefore the role of miR398/CSD in Al-stress seems to be part of the response to oxidative stress generated by different stimuli in most plant species.

Data presented in this work form a basis for further analysis leading to demonstration of specific roles of candidate common bean miRNAs in the response of common bean roots/nodules to Al-stress, using genetic approaches. Though common bean mutant collections are not yet available, reverse genetic approaches in composite bean plants, with transgenic hairy roots and nodules offer a suitable alternative. Our group in fact demonstrated the role of miR399 in phosphorus deficient bean roots using a reverse genetic approach (Valdés-López et al., 2008). Studies aimed at analysis of specific miRNA functions in response to Alt stress in SNF bean plants are in progress.

## Author Contributions

AM-S conceived and performed experiments, interpreted data and contributed to the drafting of the manuscript. LN conceived experiments, gave experimental advice and contributed to the drafting of the manuscript. AL performed experiments. GH conceived and supervised the whole project and wrote the manuscript.

## Conflict of Interest Statement

The authors declare that the research was conducted in the absence of any commercial or financial relationships that could be construed as a potential conflict of interest.

## Footnotes

1 http://www.phytozome.net, v1.0

2 http://www.phytozome.net, v1.0
